# Pediatric Features of Genetic Predisposition to Polycystic Ovary Syndrome

**DOI:** 10.1210/clinem/dgad533

**Published:** 2023-09-10

**Authors:** Jia Zhu, Anders U Eliasen, Izzuddin M Aris, Sara E Stinson, Jens-Christian Holm, Torben Hansen, Marie-France Hivert, Klaus Bønnelykke, Rany M Salem, Joel N Hirschhorn, Yee-Ming Chan

**Affiliations:** Division of Endocrinology, Boston Children's Hospital, Boston, MA 02115, USA; Programs in Metabolism and Medical and Population Genetics, The Broad Institute of MIT and Harvard, Cambridge, MA 02142, USA; Department of Pediatrics, Harvard Medical School, Boston, MA 02115, USA; Copenhagen Prospective Studies on Asthma in Childhood Research Center (COPSAC), Copenhagen University Hospital, Herlev-Gentofte, Copenhagen 2820, Denmark; Department of Health Technology, Section for Bioinformatics, Technical University of Denmark, Kongens Lyngby 2800, Denmark; Division of Chronic Disease Research Across the Lifecourse, Department of Population Medicine, Harvard Medical School, Harvard University and Harvard Pilgrim Health Care Institute, Boston, MA 02215, USA; Novo Nordisk Foundation Center for Basic Metabolic Research, Faculty of Health and Medical Sciences, University of Copenhagen, Copenhagen 2200, Denmark; Novo Nordisk Foundation Center for Basic Metabolic Research, Faculty of Health and Medical Sciences, University of Copenhagen, Copenhagen 2200, Denmark; The Children's Obesity Clinic, Accredited European Centre for Obesity Management, Department of Pediatrics, Copenhagen University Hospital Holbæk, Holbæk 4300, Denmark; Novo Nordisk Foundation Center for Basic Metabolic Research, Faculty of Health and Medical Sciences, University of Copenhagen, Copenhagen 2200, Denmark; Division of Chronic Disease Research Across the Lifecourse, Department of Population Medicine, Harvard Medical School, Harvard University and Harvard Pilgrim Health Care Institute, Boston, MA 02215, USA; Diabetes Unit, Massachusetts General Hospital, Boston, MA 02114, USA; Copenhagen Prospective Studies on Asthma in Childhood Research Center (COPSAC), Copenhagen University Hospital, Herlev-Gentofte, Copenhagen 2820, Denmark; Herbert Wertheim School of Public Health and Human Longevity Science, University of California San Diego, La Jolla, CA 92093, USA; Division of Endocrinology, Boston Children's Hospital, Boston, MA 02115, USA; Programs in Metabolism and Medical and Population Genetics, The Broad Institute of MIT and Harvard, Cambridge, MA 02142, USA; Department of Pediatrics, Harvard Medical School, Boston, MA 02115, USA; Department of Genetics, Harvard Medical School, Boston, MA 02115, USA; Division of Endocrinology, Boston Children's Hospital, Boston, MA 02115, USA; Programs in Metabolism and Medical and Population Genetics, The Broad Institute of MIT and Harvard, Cambridge, MA 02142, USA; Department of Pediatrics, Harvard Medical School, Boston, MA 02115, USA

**Keywords:** polycystic ovary syndrome, PCOS, polygenic risk score, obesity

## Abstract

**Context:**

Polycystic ovary syndrome (PCOS) has historically been conceptualized as a disorder of the reproductive system in women. However, offspring of women with PCOS begin to show metabolic features of PCOS in childhood, suggestive of childhood manifestations.

**Objective:**

To identify childhood manifestations of genetic risk for PCOS.

**Methods:**

We calculated a PCOS polygenic risk score (PRS) for 12 350 girls and boys in 4 pediatric cohorts—ALSPAC (UK), COPSAC (Denmark), Project Viva (USA), and The HOLBÆK Study (Denmark). We tested for association of the PRS with PCOS-related phenotypes throughout childhood and with age at pubarche and age at peak height velocity and meta-analyzed effects across cohorts using fixed-effect models.

**Results:**

Higher PRS for PCOS was associated with higher body mass index in midchildhood (0.05 kg/m^2^ increase per 1 SD of PRS, 95% CI 0.03, 0.07, *P* = 3 × 10^−5^) and higher risk of obesity in early childhood (OR 1.34, 95% CI 1.13, 1.59, *P* = .0009); both persisted through late adolescence (*P* all ≤.03). Higher PCOS PRS was associated with earlier age at pubarche (0.85-month decrease per 1 SD of PRS, 95% CI −1.44, −0.26, *P* = .005) and younger age at peak height velocity (0.64-month decrease per 1 SD of PRS, 95% CI −0.94, −0.33, *P* = 4 × 10^−5^).

**Conclusion:**

Genetic risk factors for PCOS are associated with alterations in metabolic, growth, and developmental traits in childhood. Thus, PCOS may not simply be a condition that affects women of reproductive age but, rather, a possible manifestation of an underlying condition that affects both sexes starting in early life.

Polycystic ovary syndrome (PCOS) is a common polygenic disorder in women of reproductive age that is characterized by ovulatory dysfunction (irregular menstrual periods) and hyperandrogenism (increased production/action of androgens such as testosterone) and is often associated with cardiometabolic dysfunction (eg, obesity, insulin resistance, and abnormal lipid levels). The pathogenesis of PCOS remains incompletely understood, and emerging evidence suggests that the underlying biological mechanisms may not be limited to women of reproductive age.

Both female and male offspring of women with PCOS show evidence of cardiometabolic dysfunction, including higher risk of obesity, insulin resistance, and dyslipidemia in childhood (1, 2). A proposed childhood precursor to PCOS is premature adrenarche, a common but poorly understood condition with early production of androgens from the adrenal glands (3). Girls with premature adrenarche often have a maternal history of PCOS and are themselves at increased risk for metabolic dysregulation, suggesting shared causal factors across these conditions (4, 5). Thus, PCOS may not simply be a condition of female reproduction but, rather, in some cases, a manifestation of an underlying disorder affecting cardiometabolic function and androgen production/action that affects both sexes from early life. Understanding these early physiological alterations in individuals at risk for PCOS could pave the way for targeted efforts to prevent, halt, and reverse the progression toward PCOS and its related conditions in childhood and adulthood.

We previously used a polygenic risk score (PRS) to estimate an individual's genetic propensity or risk for PCOS. After confirming that this PCOS PRS is associated with PCOS in women, we found that the PRS was also associated with increased risk for obesity, type 2 diabetes, coronary artery disease, and androgenic alopecia in adult men, further supporting the concept that PCOS is a manifestation of an underlying disorder that affects both sexes and also demonstrating that the PCOS PRS can be used as a proxy for this underlying disorder (6, 7). Thus, greater genetic risk for PCOS appears to have clinical and biological implications in adult women and men, beyond a clinical diagnosis of PCOS. In this study, we assessed whether features of this underlying disorder affecting cardiometabolic function and androgen production/action in adulthood could emerge in childhood. We used the PRS for PCOS to identify metabolic, growth, and developmental manifestations of this underlying disorder in childhood and adolescence in multiple pediatric cohorts (7).

## Materials and Methods

### Study Cohorts

We examined the association between genetic risk of PCOS on cardiometabolic and androgenic outcomes in four independent cohorts with data from birth until young adulthood. We grouped data into the following four age ranges: early childhood (2.5 ≤ age <6.5 years), midchildhood (6.5 ≤ age <11.5 years), early adolescence (11.5 years ≤ age <16.0 years), and late adolescence (16.0 ≤ age ≤20.0 years). Detailed characteristics for each cohort and methods for ascertainment of outcomes are reported in Table 1 and elsewhere (Table S1, and additional material (8)). Ethics approval was granted by each study's committee and review boards, and informed consent/assent was obtained from all participants and their parents/guardians, if appropriate.

**Table 1.** Characteristics of traits in childhood and adolescence across cohorts

Cohort								
Time periods	ALSPAC		COPSAC		Project Viva		The HOLBÆKK Study	
	n	Mean ± SD	n	Mean ± SD	n	Mean ± SD	n	Mean ± SD
Early childhood (2.5 ≤ age <6.5 years)								
BMI Z-score	812	0.33 ± 0.92	868	0.29 ± 0.85	404	0.65 ± 0.96	87	0.20 ± 0.90
Fat mass index (kg/m^2^)	—	—	—	—	—	—	2	3.86 ± 0.34
Lean mass index (kg/m^2^)	—	—	—	—	—	—	2	11.90 ± 0.78
HDL cholesterol (mmol/L)	494	1.93 ± 0.77	447	1.42 ± 0.32	—	—	87	1.49 ± 0.31
LDL cholesterol (mmol/L)	—	—	447	2.07 ± 0.55	—	—	87	2.23 ± 0.54
Triglycerides (mmol/L)	—	—	447	1.01 ± 0.51	—	—	87	0.60 ± 0.25
HOMA-IR*^a^*	—	—	—	—	—	—	80	1.11 ± 0.53
Midchildhood (6.5 ≤ age <11.5 years)								
BMI Z-score	5438	0.37 ± 1.00	830	0.15 ± 0.93	512	0.41 ± 1.06	877	0.27 ± 1.05
Fat mass index (kg/m^2^)	5920	4.29 ± 2.38	480	3.79 ± 1.37	410	4.22 ± 1.64	105	5.26 ± 2.45
Lean mass index (kg/m^2^)	5920	12.57 ± 0.98	480	12.57 ± 1.15	410	12.75 ± 1.20	105	13.12 ± 1.86
HDL cholesterol (mmol/L)	4747	1.52 ± 0.30	417	1.56 ± 0.31	317	1.46 ± 0.35	856	1.57 ± 0.32
LDL cholesterol (mmol/L)	785	2.08 ± 0.62	161	2.26 ± 0.51	—	—	856	2.17 ± 0.59
Triglycerides (mmol/L)	785	0.75 ± 0.27	417	0.73 ± 0.30	317	0.67 ± 0.30	856	0.62 ± 0.30
HOMA-IR*^a^*	806	1.51 ± 1.53	—	—	285	1.66 ± 1.31	848	1.91 ± 1.24
Early adolescence (11.5 years ≤ age <16.0 years)								
BMI Z-score	5065	0.32 ± 1.13	309	0.04 ± 1.12	446	0.39 ± 1.18	685	0.29 ± 1.05
Fat mass index (kg/m^2^)	4993	5.14 ± 2.96	—	—	344	6.08 ± 2.67	79	5.25 ± 2.63
Lean mass index (kg/m^2^)	4993	14.15 ± 1.51	—	—	344	14.51 ± 1.80	79	14.86 ± 1.86
HDL cholesterol (mmol/L)	3054	1.28 ± 0.29	307	1.46 ± 0.31	313	1.44 ± 0.36	665	1.48 ± 0.33
LDL cholesterol (mmol/L)	3054	2.09 ± 0.55	306	2.24 ± 0.67	—	—	665	2.05 ± 0.62
Triglycerides (mmol/L)	3054	0.84 ± 0.36	306	1.10 ± 0.61	313	0.81 ± 0.37	665	0.74 ± 0.32
HOMA-IR*^a^*	—	—	—	—	313	2.96 ± 1.82	674	3.07 ± 1.35
Late adolescence (16.0 ≤ age ≤20.0 years)								
BMI Z-score	3991	0.39 ± 1.17	309	0.35 ± 1.09	301	0.46 ± 1.07	350	0.40 ± 0.95
Fat mass index (kg/m^2^)	3835	6.30 ± 3.71	—	—	262	7.89 ± 3.25	36	6.47 ± 3.50
Lean mass index (kg/m^2^)	3835	15.37 ± 2.14	—	—	262	19.97 ± 3.77	36	15.73 ± 2.02
HDL cholesterol (mmol/L)	2731	1.27 ± 0.30	340	1.21 ± 0.26	145	1.39 ± 0.34	343	1.41 ± 0.32
LDL cholesterol (mmol/L)	2731	2.10 ± 0.60	297	2.11 ± 0.62	—	—	343	2.08 ± 0.60
Triglycerides (mmol/L)	2731	0.84 ± 0.36	340	0.97 ± 0.44	145	0.74 ± 0.33	343	0.79 ± 0.32
HOMA-IR*^a^*	—	—	—	—	140	2.27 ± 1.31	348	2.49 ± 1.15
Other								
Age at pubarche (y)	3403	11.0 ± 1.4	—	—	—	—	226	11.0 ± 1.8
Age at peak height velocity (y)	4737	12.6 ± 1.3	—	—	498	12.26 ± 1.27	—	—

Abbreviations: BMI, body mass index; HDL, high-density lipoprotein; HOMA-IR, homeostasis model assessment for insulin resistance; LDL, low-density lipoprotein; y, year.

^*a*^Units of HOMA-IR: (insulin mU/L × glucose mmol/L × 1/22.5) or (insulin μU/mL × fasting glucose mg/dL × 1/405).

The Avon Longitudinal Study of Parents and Children (ALSPAC) is a population-based birth cohort in the United Kingdom that consists of over 14 000 mother–child pairs (9, 10). Of these initial pregnancies, 13 988 children who were alive at 1 year have been followed with regular questionnaire- and clinic-based assessments, including anthropometric, biochemical, and radiographic measures. At the time of analysis, there were 8927 unrelated individuals of European ancestry with genotype data available.

The Copenhagen Prospective Studies on Asthma in Childhood (COPSAC)_2000_ and COPSAC_2010_ are 2 prospective mother–child cohorts, with children followed at scheduled clinical visits from birth until adulthood (11, 12). The combined cohorts comprise 912 children with available genotype and phenotype information.

Project Viva is an ongoing study of prenatal and perinatal influences on maternal, fetal, and child health in eastern Massachusetts (13). Anthropometric, biological, and radiographic data were collected from participants during early childhood (mean ± SD age 3.2 ± 0.2 years), midchildhood (7.9 ± 0.6 years), early adolescence (13.2 ± 0.9 years), and late adolescence (18.0 ± 1.0 years). At the time of analysis, there were 512 unrelated individuals of European ancestry with genotype data available.

The population-based control group of The HOLBÆK Study, previously referred to as The Danish Childhood Obesity Biobank, is a cross-sectional study of individuals recruited from schools within Zealand, Denmark (14, 15). Anthropometrics, body composition, and biochemical measurements were collected from participants at the time of study enrollment. At the time of analysis, there were 1999 unrelated individuals of European ancestry with genotype data.

### Study Outcomes

For all cohorts, body mass index (BMI) Z-scores for age and sex were calculated using population-based references. Obesity was defined as a BMI at or greater than 95th percentile. Fat and lean mass were measured using whole-body dual energy x-ray absorptiometry scans, and indices were calculated as kilograms per square meter. Insulin resistance was calculated using the homeostasis model assessment for insulin resistance (HOMA-IR). Lipids were obtained in the fasting state.

For androgenic outcomes, age at pubarche was derived from responses to questionnaires in ALSPAC and The HOLBÆK Study. Age at peak height velocity was derived using superimposition by translation and rotation (SITAR) mixed effects growth curve analysis in ALSPAC and Project Viva (16, 17).

### PCOS Polygenic Risk Score Calculation

We used PRS-CS software to calculate a PCOS PRS using summary statistics from the largest published genome-wide association study (GWAS) meta-analysis for PCOS, which included 113 238 women of European ancestry (18). PRS-CS is a computational method that utilizes a Bayesian approach to weight the effect size of each variant by the *P* value or strength of its association in GWAS, which allows inclusion of all variants beyond just the genome-wide significant variants (19). Thus, using the PRS-CS method, we were able to include 1 065 069 genetic variants, which significantly improved the predictive value of our PCOS PRS compared with other methods as previously shown (6, 7). We further modified the PRS-CS method to incorporate probabilistic genotype dosage from imputation (using PRSice-2 software (20) solely for calculating the PRS, without further clumping or *P* value thresholding). This PRS method has been optimized to predict the PCOS phenotype (defined by self-reported diagnoses and/or ICD-9/10 codes for irregular menses and hyperandrogenism), is strongly associated with PCOS in women in the UK Biobank (*P* = 3 × 10^−5^), and has been further validated in women in the Estonian Biobank as previously described (6, 7). To facilitate interpretation, the resulting raw PRS was scaled to a mean of 0 and an SD of 1 for each cohort. For instance, a PCOS PRS of +1.7 indicates the PRS is 1.7 SDs above the mean for the cohort.

### Statistical Analysis

For each cohort, we performed linear and logistic regression analyses to assess for associations between the PCOS PRS and the outcomes of interest for each age group (early childhood, midchildhood, early adolescence, and late adolescence), adjusting for age, age squared, and genotype batches (if any). To control for potential population stratification (ie, differences in allele frequencies between populations or subpopulations), we derived the first 10 genetic principal components of ancestry and included them as additional covariates in all regression analyses. To assess for the effect of sex in the relationship between the PCOS PRS and the outcome of interest, we (1) controlled for sex as a covariate, (2) performed sex-stratified analyses, and (3) modeled sex as an interaction term. Overall effect-size estimates for each age group, including sex-specific effects, were calculated by meta-analyzing cohort-specific estimates using an inverse variance–weighted fixed-effect model. Heterogeneity between studies was measured using *I^2^* statistics, with <25% indicating low, 25% to 75% moderate, and >75% considerable heterogeneity.

To determine the effect of the PCOS PRS on the rate of change in BMI Z-score, we used linear spline mixed-effect models and longitudinal BMI Z-scores from age 7 years (first focus study assessment clinic) to 17 years in the ALSPAC cohort. We identified 2 knot points at 10 and 13 years that captured the trajectory of BMI Z-score change (Table S2 and Fig. S3 (8)). We grouped participants by PCOS PRS quintile and examined BMI Z-score trajectories according to quintile. In a single model, we included the PCOS PRS quintiles as a fixed effect and as an interaction with each of the 3 linear slopes in the linear spline models and as an interaction with sex. We also included the first 10 genetic principal components as fixed effects. We included a random intercept and slopes to account for repeated BMI Z-score measurements in each participant. For each PCOS PRS quintile, we used the linear spline model to predict BMI Z-scores from age 7 to 17 years and plot its trajectory for each participant. We provide additional details of this analysis elsewhere (8).

To account for the contribution of maternal genetic risk factors for PCOS to the association of the child's genetic risk for PCOS and outcomes throughout childhood in the ALSPAC cohort, we conducted a linear regression of the child's PCOS PRS on the mother's PCOS PRS and used the residuals from this regression to assess associations with outcomes.

All statistical analyses were conducted using R v3.5.0. The R package “meta” was used for all meta-analyses (21), and “lme4” was used to construct linear spline mixed-effect models (22).

## Results

### Association Between Genetic Risk for PCOS and BMI in Childhood

**Figure 1.**
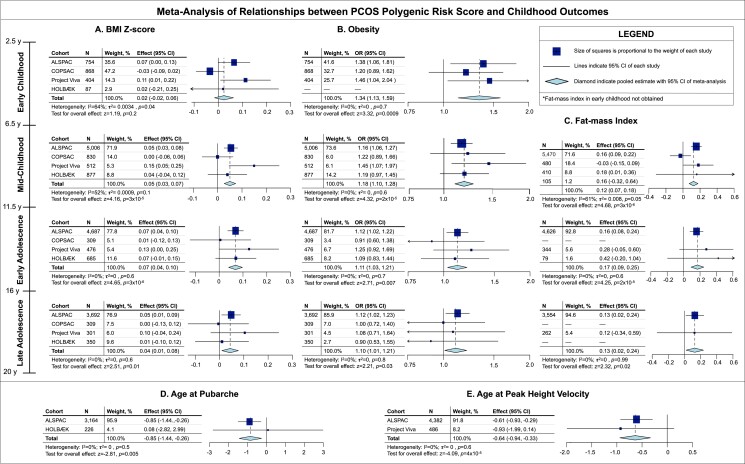
Relationships of PCOS genetic risk score (PRS) with BMI Z-score (A), obesity (B), and fat mass index (kg/m^2^) (C) throughout childhood and with ages at pubarche (months) (D), and peak height velocity (months) (E). N, number of participants; OR, odds ratio; BMI, body mass index; y, years.

We observed that a higher PCOS PRS (indicating greater genetic predisposition to PCOS) was associated with higher BMI Z-score starting in the midchildhood period (0.05 SDS increase for every 1 SD increase in the PRS, 95% CI 0.03-0.07, *P* = 3 × 10^−5^; Fig. 1A). For instance, on average, individuals with a PCOS PRS of 1.5 have a 1.5 × 0.05 = 0.075 higher BMI Z-score than individuals with a PRS of 0. The association between a higher PCOS PRS and higher BMI Z-score persisted through late adolescence with comparable effect sizes (*P* all ≤.01, I^2^ = 0-52%; low to moderate heterogeneity). We also observed that a higher PCOS PRS was associated with increased odds of obesity in early childhood (OR 1.34, 95% CI 1.13-1.59, *P* = .0009; Fig. 1B). This association between a higher PCOS PRS and increased odds of obesity persisted through late adolescence (*P* all ≤ .03, I^2^ = 0%; low heterogeneity), though the effect appeared to attenuate in early and late adolescence (Fig. 1B).

### Association Between Genetic Risk for PCOS and Body Composition in Childhood

BMI is an indirect measure of adiposity but has some limitations in children (23, 24). Thus, we directly examined the association between the PCOS PRS and body composition as determined by whole-body dual energy x-ray absorptiometry scans where available. We observed increases in fat mass index (FMI) ranging from 0.12 to 0.17 kg/m^2^ for every 1 SD increase in the PRS during childhood and adolescence (*P* all ≤ .02, I^2^ = 0-61%; low to moderate heterogeneity; Fig. 1C). For reference, the mean FMI in midchildhood in the ALSPAC cohort fis 4.29 kg/m^2^ (Table 1), so an increase of 0.12 kg/m^2^ corresponds to a 2.8% increase above the mean FMI. We observed an association between a higher PCOS PRS and higher lean-mass index in early adolescence (0.05 kg/m^2^ increase for every 1-SD increase in the PRS, 95% CI 0.01, 0.09, *P* = .01, I^2^ = 68%; moderate heterogeneity; Fig. S1B (8)). We did not identify any significant associations between the PCOS PRS and lean mass index in midchildhood or late adolescence (*P* > .05).

### Association Between Genetic Risk for PCOS and Cardiometabolic Risk Factors in Childhood

Early manifestations of cardiometabolic dysfunction can include insulin resistance and dyslipidemia in offspring of women with PCOS (1, 2). Thus, we assessed the association between genetic risk for PCOS and these metabolic traits. We did not observe significant associations between the PCOS PRS and high-density lipoprotein (HDL) cholesterol, fasting low-density lipoprotein (LDL) cholesterol, or fasting triglycerides, or with insulin resistance as assessed by HOMA-IR (Fig. S1A and S2 (8)).

### Sex-Specific Associations Between Genetic Risk for PCOS and Cardiometabolic Phenotypes in Childhood

**Figure 2.**
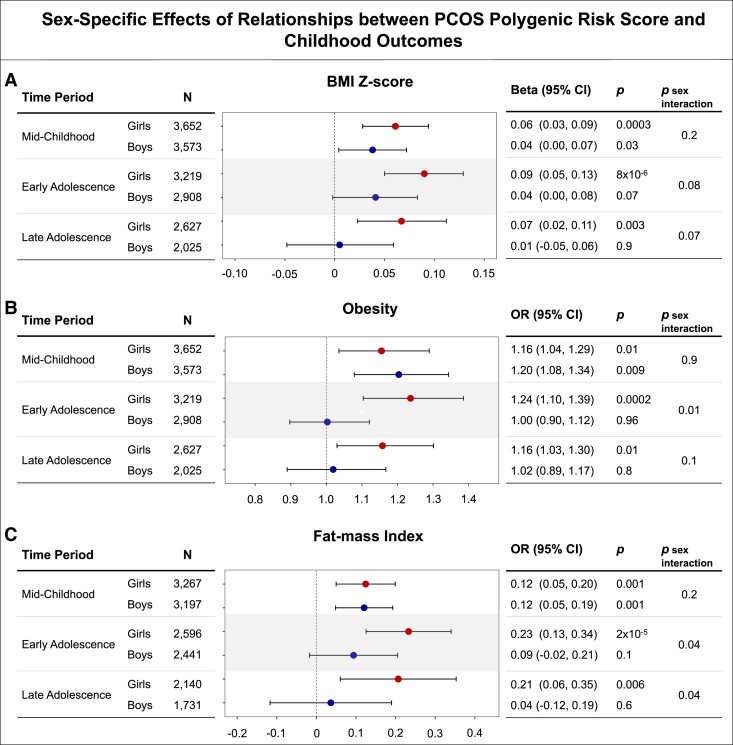
Effects of sex on relationships of PCOS genetic risk score (PRS) with BMI Z-score (A), obesity (B), and fat mass index (kg/m^2^) (C) throughout childhood and adolescence. N, number of participants; OR, odds ratio; BMI, body mass index.

To assess sex-specific associations, we performed sex-stratified analyses and modeled sex as an interaction term in the midchildhood, early adolescence, and late adolescence time periods. In midchildhood, the effects of the PCOS PRS on BMI Z-score, obesity, and FMI were comparable between girls and boys, and we observed no significant sex-specific effects (*P* for interaction term between sex and PCOS PRS all > 0.2; Fig. 2). Starting in early adolescence, a higher PCOS PRS was associated with greater odds of obesity and higher FMI in girls than in boys (*P* for interaction term between sex and PCOS PRS = .01 and .04, respectively; Fig. 2). This pattern of greater effect of the PCOS PRS in girls than in boys appeared to persist into late adolescence for FMI (*P* for sex interaction = .04), but not for obesity (*P* for sex interaction = .1). For BMI Z-scores, although sex-stratified analyses suggested greater estimated effects of the PCOS PRS on BMI Z-score in girls than in boys in both early and late adolescence, the *P* values for sex interactions were both greater than .07 (Fig. 2).

### Association Between Genetic Risk for PCOS and Age at Pubarche and Peak Height Velocity

In ALSPAC and The HOLBÆK Study, we tested the association between the PCOS PRS and age at first pubic hair (ie, pubarche), a clinical measure of androgen action. We observed that a higher PRS was associated with a younger age at pubarche (0.85 months decrease in the age at pubarche per 1 SD increase in PRS, 95% CI −1.44, −0.26, *P* = .005, I^2^ = 0%; low heterogeneity; Fig. 1). In ALSPAC and Project Viva, we also assessed the association between the PCOS PRS and age at peak height velocity, which is influenced by androgen levels, and we observed that a higher PRS was associated with a younger age at peak height velocity (β = .64 month decrease in the age at peak height velocity per 1 SD increase in PRS, 95% CI −0.94, −0.33, *P* = 4 × 10^−5^, I^2^ = 0%; low heterogeneity; Fig. 1).

Because higher BMI has been associated with both earlier age at pubarche and peak height velocity (25, 26), we additionally adjusted for prepubertal BMI Z-score at 8 years as a covariate and observed persistent associations with comparable effect sizes for pubarche (0.96 month decrease for every 1 SD increase in the PRS, 95% CI −1.61, −0.32, *P* = .003; I^2^ = 0%; low heterogeneity) and peak height velocity (0.44 month decrease for every 1 SD increase in the PRS, 95% CI −0.65, −0.23, *P* = 4 × 10^−5^, I^2^ = 0%; low heterogeneity).

We did not detect significant sex-specific effects of the PCOS PRS on age at peak height velocity or age at pubarche (*P* for interaction term between sex and PCOS PRS ≥ .2; Table S3 (8)).

### Association of Genetic Risk for PCOS With BMI Z-score Trajectories in Childhood

**Figure 3.**
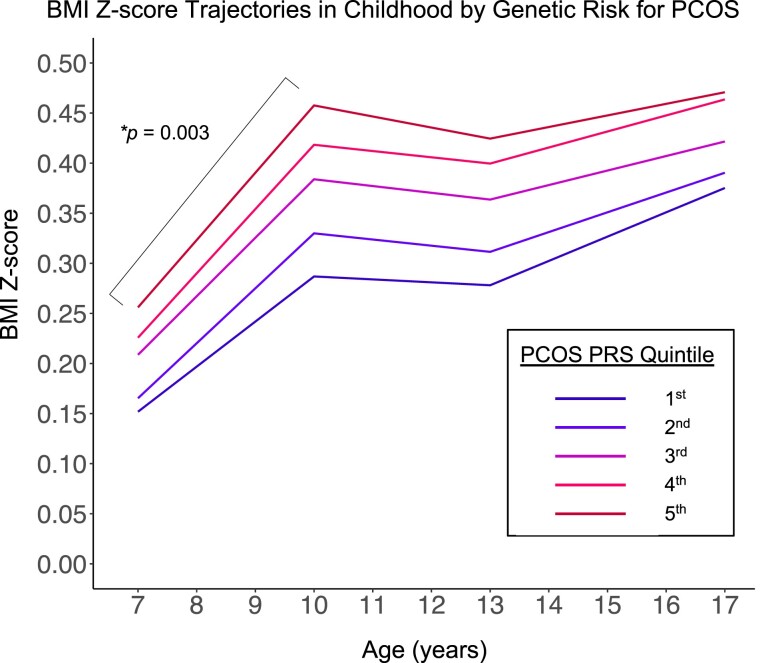
Trajectories of BMI Z-score from midchildhood to late adolescence according to quintile of PCOS polygenic risk score (PRS) among participants of the ALSPAC cohort. The model was adjusted for the first 10 genetic principal components and sex. Quintiles are ordered from lowest to highest PCOS PRS. *Bracket denotes higher rate of BMI Z-score gain for participant with a PCOS PRS in the fifth vs first quintile in the midchildhood time period.

In ALSPAC, we used longitudinal BMI Z-score data to determine if the PCOS PRS is associated with BMI Z-score trajectory in late childhood (specifically, the rate of change in BMI Z-score for measurements between 7 and 10 years). The top quintile for the PCOS PRS had a more rapid increase in BMI Z-score per year from 7 to 10 years (β = .026, 95% CI 0.009, 0.042, *P* = .003; Table 2 and Fig. 3) compared with the bottom quintile. The association of the PCOS PRS on BMI Z-score trajectory was comparable between girls and boys, with no sex-specific effect (*P* for interaction term between sex and PCOS PRS quintiles 2 through 5 compared with quintile 1 all ≥0.2).

**Table 2.** Associations of the PCOS PRS with rate of BMI z-score change throughout childhood

	7-10 years			10-13 years			13-17 years		
No. of subjects: 6972	β	95% CI	*P*	β	95% CI	*P*	β	95% CI	*P*
PCOS PRS quintile									
2nd (vs 1st)	0.015	−0.001, 0.032	.07	−.006	−0.024, 0.012	.5	−.007	−0.024, 0.011	.5
3rd	0.015	−0.002, 0.032	.08	−.002	−0.019, 0.016	.8	−.017	−0.034, 0.000	.06
4th	0.020	0.004, 0.037	.02	−.001	−0.018, 0.017	.9	−.008	−0.025, 0.009	.3
5th	0.026	0.009, 0.042	.003	−.012	−0.030, 0.006	.2	−.014	−0.031, 0.003	.1

Abbreviations: BMI, body mass index; PCOS, polycystic ovary syndrome; PRS, polygenic risk score.

### The Role of Maternal Genetic Risk for PCOS in the Association Between Child Genetic Risk for PCOS and Childhood Outcomes

To assess the possibility that the child's PCOS PRS could be acting as a surrogate for the mother's PCOS PRS, rather than affecting outcomes directly, we examined the role of the maternal PCOS PRS in the associations between the child's PCOS PRS and outcomes in 3849 mother–child pairs in the ALSPAC cohort. After regressing on the maternal PCOS PRS, the child PCOS PRS residual showed persistent significant associations with metabolic, growth, and developmental outcomes from midchildhood to late adolescence (Table 3). For instance, the association between a higher PCOS PRS and higher BMI Z-score in middle childhood (*P* = 7 × 10^−6^) retained significance using the child PCOS PRS residual (*P* = .002). Thus, a child's PCOS PRS appears to have direct effects on childhood outcomes, independent of maternal polygenic risk for PCOS.

**Table 3.** The role of maternal PCOS PRS in the associations between child PCOS PRS and childhood outcomes in ALSPAC

Association of the child PCOS PRS vs child PCOS PRS residual*^a^* and cardiometabolic outcomes												
Time periods	n	BMI Z-score			n	Obesity			n	Fat mass index		
		β	95% CI	*P*		OR	95% CI	*P*		β	95% CI	*P*
Early childhood	588				588				—			
Child PRS		.04	−0.04, 0.12	.3		1.20	0.89, 1.63	.2		—	—	—
Child PRS residual		.05	−0.035, 0.139	.2		1.31	0.93, 1.85	.1		—	—	—
Midchildhood	3861				3861				4156			
Child PRS		.07	0.04, 0.11	7 × 10^−6^		1.22	1.11, 1.36	9 × 10^−5^		.19	0.12, 0.26	9 × 10^−8^
Child PRS residual		.06	0.02, 0.10	.002		1.15	1.03, 1.30	.02		.13	0.05, 0.21	.002
Early adolescence	3829				3829				3782			
Child PRS		.08	0.04, 0.11	4 × 10^−5^		1.14	1.04, 1.26	.007		.18	0.10, 0.27	6 × 10^−5^
Child PRS residual		.07	0.02, 0.11	.002		1.13	1.01, 1.26	.03		.15	0.04, 0.25	.006
Late adolescence	2966				2966				2858			
Child PRS		.06	0.01, 0.10	.009		1.16	1.04, 1.29	.007		.18	0.06, 0.30	.003
Child PRS residual		.05	0.00, 0.10	.052		1.13	1.00, 1.28	.04		.14	0.00, 0.28	.06
Association of the child PCOS PRS vs child PCOS PRS residual^a^ and growth outcomes												
		**Age at pubarche n = 2421**				**Age at peak height velocity n = 3543**						
Child PRS		−1.04	−1.73, −0.35	.003		−0.68	−1.03, −0.32	.0002				
Child PRS residual		−0.90	−1.71, −0.09	.03		−0.63	−1.03, −0.22	.003				

Abbreviations: BMI, body mass index; n, number of mother–child pairs; PCOS, polycystic ovary syndrome; PRS, polygenic risk score.

^*a*^Adjusted for maternal PCOS PRS.

## Discussion

We found that polygenic risk for PCOS, a disorder of women of reproductive age, is associated with alterations in metabolic, growth, and developmental traits that emerge in early life and persist into adolescence in both boys and girls. These childhood manifestations of genetic liability to PCOS can be seen at ages well before the typical age range for gonadarche and adrenarche and thus provide evidence that genetic risk factors for PCOS can act independently of ovarian and adrenal androgen function. Furthermore, we identified associations between PCOS genetic risk factors and childhood phenotypes in both girls and boys with sex-specific effects for obesity and FMI (greater effects in girls vs boys) emerging in early adolescence. Thus, our results provide support that PCOS is not solely a condition that affects women of reproductive age, but rather a possible manifestation of an underlying condition affecting cardiometabolic function and androgen production/action that affects both sexes starting in early life and extending into adulthood.

Multiple mechanisms could account for the association between polygenic risk for PCOS and alterations in metabolic, growth, and developmental traits in early life. Genetic risk for PCOS could produce an antecedent in childhood that leads to PCOS in adulthood. For instance, we found associations between the PCOS PRS and childhood obesity, and higher BMI causes increased risk for PCOS (27). We did not identify associations between the PCOS PRS and other cardiometabolic risk factors associated with obesity, including fasting lipids and insulin resistance. One possibility may be that these risk factors have not yet manifested in childhood. Alternatively, PCOS could have distinct manifestations in both childhood and adulthood that could be both dependent on and independent of BMI. Supportive of this, we identified associations between polygenic risk for PCOS and younger age at pubarche and peak height velocity, both key events in childhood growth and development, and these associations persisted even after adjusting for BMI, suggesting the presence of BMI-independent biological pathways. Finally, it is also possible that both mechanisms are in play and may contribute to varying degrees from individual to individual.

While polygenic risk for PCOS is associated with alterations in metabolic traits in both girls and boys, our findings suggest that sex-specific effects (stronger effects in girls than in boys) may emerge in early adolescence. Possible mechanisms underlying these sex-specific effects of PCOS genetic risk factors include differences in the sex hormones that are made during puberty as well as the earlier timing of sex hormone production in girls compared with boys. Other possibilities include modifying genetic risk factors on the sex chromosomes, differences in gene expression, and/or complex environmental interactions that may emerge in adolescence. Future classification of the genetic variants that have sex-specific effects on PCOS-related traits will allow for identification of potential mechanistic pathways.

Polygenic risk for PCOS could have direct effects in children; alternatively, genetic risk for PCOS could have indirect effects in children through direct effects on their mothers. Indeed, an altered intrauterine environment in mothers with PCOS has been proposed to increase the risk of cardiovascular disease in offspring (1, 28). If the effects of polygenic risk for PCOS affect children indirectly, the effect of the child's PCOS PRS after adjusting for the mother's PCOS PRS would be predicted to be significantly attenuated. However, we observed persistent significant associations between the child's residual PCOS PRS and cardiometabolic and growth outcomes. Thus, a child's polygenic risk for PCOS does not appear to be merely a surrogate for maternal polygenic risk for PCOS.

Limitations of our study include the predictive power of our PCOS PRS, which is limited by the heritability of PCOS attributable to common variants and by the power of the existing GWAS meta-analysis for PCOS to detect those common variants (18). The current GWAS meta-analysis was primarily conducted in individuals of European ancestry, and genetic risk scores are less valid (with current methodologies) when applied to populations with different ancestry (29). Inclusion of cohorts with diverse ethnicities in GWAS meta-analyses will improve the accuracy of genetic risk score for PCOS in populations of all ancestral backgrounds. Despite these limitations, we were able to demonstrate significant effects of polygenic risk for PCOS on childhood outcomes. While the effects of the PCOS PRS on childhood outcomes are modest, even modest elevations of BMI Z-scores within the nonobese range in childhood and adolescence have been shown to increase risk for obesity-related disorders in adulthood, including coronary heart disease and type 2 diabetes (30, 31). An additional limitation of our study is the variability in the timepoints for available data in our four pediatric cohorts. To address this issue, we grouped participants into 4 age periods to account for important developmental timeframes and conduct cohort-wide meta-analyses. Furthermore, a strength of our study is the number of pediatric cohorts and size of each cohort with genotyping and extensive anthropometric, clinical, biochemical, and imaging data, which allowed for identification of even modest associations between polygenic risk for PCOS and childhood outcomes.

In conclusion, the results of our study suggest that genetic liability for PCOS can result in alterations in metabolic, growth, and developmental traits in both sexes starting in early life and extending into adulthood. These alterations may reflect early physiological changes of an underlying condition affecting cardiometabolic function and androgen production/action, of which PCOS is just one possible manifestation. Future dissection of the underlying molecular pathways will inform strategies to prevent, halt, and reverse these alterations before the development of PCOS and its related conditions in childhood and adulthood.

## Data Availability

Restrictions apply to the availability of all data generated during this study. Data from ALSPAC, COPSAC, Project Viva, and The HOLBÆK Study are available to researchers, and data access is governed by the respective board of directors and/or principal investigators.

## Abbreviations

BMI: body mass index
FMI: fat mass index
GWAS: genome-wide association study
HOMA-IR: homeostasis model assessment for insulin resistance
PCOS: polycystic ovary syndrome
PRS: polygenic risk score
